# Sensors Allocation and Observer Design for Discrete Bilateral Teleoperation Systems with Multi-Rate Sampling

**DOI:** 10.3390/s22072673

**Published:** 2022-03-30

**Authors:** Amir Aminzadeh Ghavifekr, Roberto De Fazio, Ramiro Velazquez, Paolo Visconti

**Affiliations:** 1 Faculty of Electrical and Computer Engineering, University of Tabriz, Tabriz 5166616471, Iran; aa.ghavifekr@tabrizu.ac.ir; 2 Department of Innovation Engineering, University of Salento, 73100 Lecce, Italy; roberto.defazio@unisalento.it; 3 Facultad de Ingeniería, Universidad Panamericana, Aguascalientes 20290, Mexico; rvelazquez@up.edu.mx

**Keywords:** teleoperation, sensor allocation, multi-rate sampling, exponential stability, LMIs

## Abstract

This study addresses sensor allocation by analyzing exponential stability for discrete-time teleoperation systems. Previous studies mostly concentrate on the continuous-time teleoperation systems and neglect the management of significant practical phenomena, such as data-swap, the effect of sampling rates of samplers, and refresh rates of actuators on the system’s stability. A multi-rate sampling approach is proposed in this study, given the isolation of the master and slave robots in teleoperation systems which may have different hardware restrictions. This architecture collects data through numerous sensors with various sampling rates, assuming that a continuous-time controller stabilizes a linear teleoperation system. The aim is to assign each position and velocity signals to sensors with different sampling rates and divide the state vector between sensors to guarantee the stability of the resulting multi-rate sampled-data teleoperation system. Sufficient Krasovskii-based conditions will be provided to preserve the exponential stability of the system. This problem will be transformed into a mixed-integer program with LMIs (linear matrix inequalities). These conditions are also used to design the observers for the multi-rate teleoperation systems whose estimation errors converge exponentially to the origin. The results are validated by numerical simulations which are useful in designing sensor networks for teleoperation systems.

## 1. Introduction

Teleoperation systems have been extensively deployed in remote and hazardous environments during the last decades. A teleoperation system consists of master and slave mechanical parts where the master manipulates the slave via a human operator. A detailed survey of common definitions, applications, and developments of teleoperation systems is provided in [1,2].

Time delays mainly caused by data transfer across communication channels are considered, representing one of the most significant challenges in teleoperation systems since even a minor time delay can destabilize the entire teleoperation system. Motivated to solve the instability caused by time delays, a wide range of approaches have been proposed even in the recent literature for a continuous-time model of the bilateral systems. In [3], a modified wave variable compensation has been utilized to passivate the delayed communication channel. In [4], a robust controller based on a state observer is implemented on a haptic device and an asymmetric teleoperation system. The results demonstrate better tracking performance for force signals. A modified robust adaptive controller with a novel adaptive torque observer for nonlinear teleoperation systems has been proposed in [5] to overcome time-varying delays, external disturbances and uncertainties in the system dynamics. This structure is relaxed from acceleration measurement and does not require force sensors. In [6], the admittance control is applied to study the phase transition between the constraint and unconstraint motions in the teleoperator system with haptic feedback. Its stability proof is provided by hybrid systems theory and Lyapunov–Krasovskii approach. A modified motion prediction method is proposed in [7], based on a state observer with a cascade architecture. Under an adequate set of assumptions for the existence of uncertainties, the prediction errors remain bounded. These results, properly extended, have been applied to a scaled four-channel scheme under time-varying delays in [8]. Also, a novel adaptive-observer-based scaled controller for four-channel systems is presented in [9] to overcome asymmetric time-varying delays.

Besides these model-based methods, data-driven approaches have also been utilized in teleoperation systems. In [10], an adaptive neural network-based controller is designed in the existence of external disturbances, constant time delay, and internal frictions. Also, in [11], a truncated quantile critics reinforcement learning-based integrated structure is presented, leading to the quantifiable training feedback in a teleoperation system. A radial basis function neural network (RBFNN) is applied in [12] to estimate dynamic uncertainties in the existence of the actuator backlash-like hysteresis and time-varying delays.

Obviously, all of the studies above mainly have focused on the continuous-time teleoperation systems, neglecting the handling of some practical parameters such as data-swap, the sampling rate effect, and update rates of actuators on the system’s stability. However, this investigation can be instructive in experimental studies such as [13], which proposes an open-source scheme of a wearable 7-DOF wireless operator arm motion-tracking system for teleoperator systems or in [14], where a force tactile feedback approach for finger tracking systems is presented.

Although continuous-time teleoperator systems have received much attention, there are far fewer studies for sampled-data counterparts. However, some discrete-nature controllers have been reported for teleoperation systems in recent years. A review of discrete-time control methods for sampled-data teleoperator systems has been presented in [15]. Also, a detailed comparison between the behavior of analog and sampled-data teleoperation systems is investigated in [16]. In [17], the data transfer from multiple slave robots to the master robot is regulated using the round-robin (RR) scheduling algorithm for a class of sampled-data teleoperation systems. In [18], an original method to teleoperate a discrete hyper-redundant robots is presented by utilizing mixed reality to carry out complex tasks in remote environments with more accuracy. In [19], a modified discrete sliding mode controller is proposed via a new reaching law to overcome the chattering phenomena besides disturbance rejection by utilizing an adaptive extended state observer.

A significant deficiency in previous literature on discrete-time teleoperations systems is that all transmitted signals are sampled with the same sampling rate. However, it is not a sensible assumption in the teleoperation systems due to the isolation of the master and slave robots, which may have different hardware restrictions; thus, a multi-rate approach has been proposed in this paper. In the multi-rate architecture, data are collected through numerous sensors with various sampling rates. The necessity of multi-rate sampling is not just due to different phenomena in the system. Sometimes utilizing different methods to sense the same phenomena provides unequal sampling rates in the system. As an instance in our presented study, the position signal of the master robot can be measured by either encoder, potentiometer or even a camera via image processing. Even if we relinquish applying different sensors, variable delays and data drop out in communication channels lead to different data arriving times for the controllers.

Stability analysis of the multi-rate systems and designing sampled-data controllers have received wide attention in recent years [20,21,22,23]. In an extension to our previous study in [24], which proposes Krasovskii-based conditions in the form of LMIs (Linear Matrix Inequalities) to stabilize the linear multi-rate teleoperation systems, this study focuses on sensor allocation with analyzing exponential stability. It is presumed that a continuous-time controller stabilizes the teleoperator system. The goal is to assign each position and velocity signals to sensors with different sampling rates and divide the state vector across sensors so that the multi-rate system remains exponentially stable. Different approaches for dividing the state vector across sensors can lead to stable or unstable systems. Thus, Krasovskii-based conditions satisfy exponential stability. Also, these conditions are employed to design observers on both sides of the teleoperator system. This problem will be transformed into a mixed-integer program with LMIs. The results of this paper are useful in designing sensor networks for teleoperation systems.

The remainder of this study is organized as follows. In Section 2, preliminaries and sampled-data model of the teleoperator system with multi-rate sampling approach are presented. The exponential stability analysis for sensor partitioning and observer design are provided in Section 3 and Section 4, whereas in Section 5, these results are validated by numerical simulations. In Section 6 and Section 7, the results are reported and concluding remarks stated.

## 2. Proposed Sampled-Data Model with Multi-Rate Architecture for Teleoperation System

**Figure 1:**
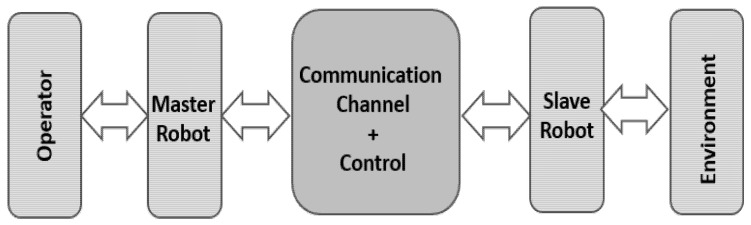
The general schematic of the teleoperation system.

In a bilateral teleoperation system, when a human manipulates the master robot, the generated motion signals are relayed to the slave robot via a wireless network, and the slave robot subsequently performs the same action. The general schematic of the teleoperation system is depicted in Figure 1.

### 2.1. Continuous-Time Model

In the proposed model for robotic manipulators as the master and slave subsystems, position and velocity signals have been considered as states of the system with this limitation that there is no gravitational force and friction. Also, it is presumed that the force from the human operator applied to the master robot can be modeled as a passive mass-spring-damper system. The exogenous force input exerted by the operator is presumed to be zero.

In the absence of frictions and external disturbances and assuming two degrees of freedom robots as master and slave subsystems, the linearized dynamics can be derived as:

(1) $$\begin{array}{l} M_{m}{\ddot{q}}_{m}+D_{m}{\dot{q}}_{m}+S_{m}q_{m}=F_{m}+F_{h}\\ M_{s}{\ddot{q}}_{s}+D_{s}{\dot{q}}_{s}+S_{s}q_{s}=-F_{e}-F_{s} \end{array}$$

where subscripts *m* and *s* are notifications for master and slave robots. M, D and, S are used for mass, damping, and stiffness of robots, respectively. Position and velocity signals are mentioned by q and $\dot{q}.$ $F_{h}$ and $F_{e}$ are imposed forces to the master and slave robots by operator and environment, respectively. $F_{m}$ and $F_{s}$ denote control signals.

The slave robot can be in a free motion or in contact with the environment. Let define the constant parameter κ=1 for contact case and κ=0 for free motion. The state-space equation can be stated as:

(2) $$\left[\begin{array}{l} {\dot{q}}_{m}\\ {\ddot{q}}_{m}\\ {\dot{q}}_{s}\\ {\ddot{q}}_{s} \end{array}\right]=\left[\begin{array}{cc} A_{11} & \underline{0}\\ \underline{0} & A_{22} \end{array}\right]\left[\begin{array}{l} q_{m}\\ {\dot{q}}_{m}\\ q_{s}\\ {\dot{q}}_{s} \end{array}\right]+\left[\begin{array}{l} B_{1}\\ B_{2} \end{array}\right]\left[F_{m}\;\;F_{s}\right]$$

where

(3) $$\begin{array}{l} A_{11}=\left[\begin{array}{cc} 0 & 1\\ -\frac{S_{m}+S_{h}}{M_{m}+M_{h}} & \frac{S_{m}+S_{h}}{M_{h}+M_{m}} \end{array}\right]\\ A_{22}=\left[\begin{array}{cc} 0 & 1\\ -\frac{S_{s}+\kappa S_{e}}{M_{s}+\kappa M_{e}} & -\frac{D_{s}+\kappa D_{e}}{\kappa M_{e}+M_{s}} \end{array}\right] \end{array}$$

and

(4) $$B_{1}=\left[\begin{array}{c} 0\\ \frac{1}{M_{m}+M_{h}} \end{array}\right],B_{2}=\left[\begin{array}{c} 0\\ \frac{1}{M_{s}+M_{e}} \end{array}\right]$$

where subscripts *e* and *h* are used for environment, and human operator, respectively.

### 2.2. Sampled-Data Model

Assuming the constant sampling period $t_{s}$ for all transmitted signals, the sampled-data counterparts of the aforementioned teleoperation system can be derived as:

(5) $$\begin{array}{l} q_{m}(k+1)=A_{d}q_{m}(k)+B_{d}F_{m}(k)+\int_{kt_{k}}^{(k+1)t_{k}}e^{\left|(k+1)t_{k}-\tau \right|A}BF_{h}(\tau )d\tau \\ q_{s}(k+1)=A_{d}q_{s}(k)+B_{d}F_{s}(k) \end{array}$$

where

(6) $$A_{d}=e^{At_{k}},B_{d}=\int_{kt_{k}}^{(k+1)t_{k}}e^{\left[(k+1)t_{k}-\tau \right]A}Bd\tau =\int_{0}^{t_{k}}e^{A\tau }Bd\tau$$

**Figure 2:**
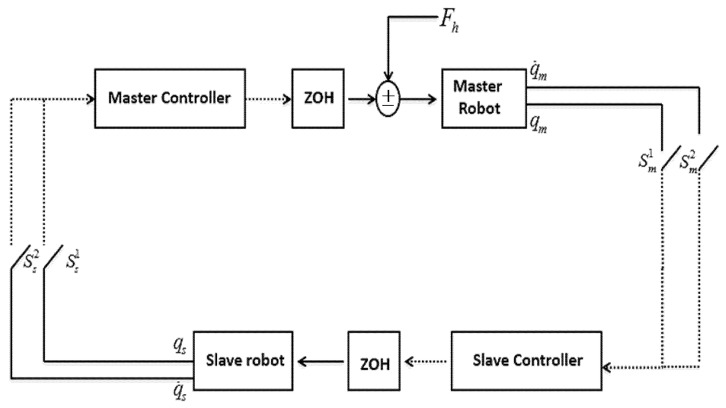
Schematicofsampled-databilateral teleoperationsystemwitha multi-rate sampling approach.

However, due to the reasons provided in the introduction section, a multi-rate approach is utilized in this study. Without losing the generality of the problem, the sampling rates are assumed to be the same for position signals and differ with velocity sensors in both master and slave robots sides. For this purpose, let us define the state vector as: $q={\left[q_{m}^{T}\;{\dot{q}}_{m}^{T}\;q_{s}^{T}\;{\dot{q}}_{s}^{T}\right]}^{T}$ and assume that both sides are sampled by varying sampling rates, represented by $T_{\chi }^{1}$ and $T_{\chi }^{2}$.(χ=m for the master robot and χ=s for the slave robot). Thus, the sampling periods can be named by $t_{\chi k}^{1}$ and $t_{\chi k}^{2}.$ Also, for zero-order holds (ZOHs), which are indicated by $Z_{\kappa }$, the refresh rates are donated by $z_{\chi k}$. The schematic architecture for the proposed sampled-data bilateral system with multi-rate sampling, and 2 DOF robotic manipulators in slave and master robots sides is depicted in Figure 2.

**Assumption** **1.** *For samplers*$\varepsilon_{s}>0$*exists which satisfies *$\varepsilon_{s}<t_{\chi_{k+1}}^{1}-t_{\chi_{k}}^{1}$*and*$\varepsilon_{s}<t_{\chi_{k+1}}^{2}-t_{\chi_{k}}^{2}$.

**Assumption** **2.** *For actuators*$\varepsilon_{z}>0$*exists which satisfies*$\varepsilon_{z}<z_{\chi_{k+1}}^{1}-z_{\chi_{k}}^{1}$.

These assumptions mention that sampling and updating operations cannot occur twice at the same period. The time that has passed since the last sampling point is called the elapsed time and calculated as:

(7) $$\eta_{\chi }^{1}\left(t\right)≜t-t_{\chi^{k}}^{1},\;\forall t\in \left[t_{k}^{1},\;t_{k+1\;}^{1}\right)\;\eta_{\chi }^{2}\left(t\right)≜t-t_{\chi^{k}}^{2},\;\forall t\in \left[t_{k}^{2},\;t_{k+1\;}^{2}\right)$$

The same definitions can be considered for actuators:

(8) $$\eta_{z}\left(t\right)≜t-z_{k},\;\forall t\in \left[z_{k},\;z_{k+1}\right]$$

This last shows the elapsed time for ZOHs. The maximum sampling and updating periods that satisfy the stability are defined by:

(9) $$\begin{array}{l} \sigma_{j}^{1}=\sup(\eta_{\chi }^{1}(t))=\sup(t_{\chi_{k+1}}^{1}-t_{\chi_{k}}^{1})\\ \sigma_{j}^{2}=\sup(\eta_{\chi }^{2}(t))=\sup(t_{\chi_{k+1}}^{2}-t_{\chi_{k}}^{2}) \end{array}\;\sigma_{z}=\sup(\eta_{z}(t))=\sup(z_{k+1}-z_{k})$$

The duration of data transmission time from sensors to ZOHs can be calculated as:

(10) $$\begin{array}{l} \eta_{\chi_{tz}}^{1}(t)=t-z_{\chi_{k}}+\eta_{\chi }^{1}(z_{k}),\forall t\in [z_{k},z_{k+1}]\\ \eta_{\chi_{tz}}^{2}(t)=t-z_{\chi_{k}}+\eta_{\chi }^{2}(z_{k}),\forall t\in [z_{k},z_{k+1}] \end{array}$$

**Remark** **1.** *Unlike*$\eta_{\chi }(t)$*and*$\eta_{z}(t)$*, the function*$\eta_{\chi_{sz}}(t)$*can be assigned by non-zero amounts at its discontinuities*. *Thus*:

(11) $$0\leq \eta_{\chi_{sz}}(t)<\sigma_{\chi }+\sigma_{z}=\sigma_{\chi z}$$

*where*$\sigma_{\chi z}$*indicates the upper bound for data transmission time from sensors to ZOHs*.

In the literature, proportional-derivative (PD) continuous-time controllers are proposed to provide a stable and transparent teleoperation system. The sampled-data counterpart of these controllers are:

(12) $$\begin{array}{l} F_{m}(k+1)=-K_{v}({\dot{q}}_{m}(k)-{\dot{q}}_{s}(k))-K_{P}(q_{m}(k)-q_{s}(k))\\ F_{s}(k+1)=-K_{v}({\dot{q}}_{s}(k)-{\dot{q}}_{m}(k))-K_{P}(q_{s}(k)-q_{m}(k)) \end{array}$$

where $K_{p}$ and $K_{v}$ are constant positive elements.

Applying the input delay approach [24], control laws are rewritten as:

(13) $$\begin{array}{l} F_{m}(t)=-K_{v}({\dot{q}}_{m}(t-\eta_{\chi_{sz}}^{2})-{\dot{q}}_{s}(t-\eta_{\chi_{sz}}^{2}))-K_{P}(q_{m}(t-\eta_{\chi_{sz}}^{1})-q_{s}(t-\eta_{\chi_{sz}}^{1}))\\ F_{s}(t)=-K_{v}({\dot{q}}_{2}(t-\eta_{\chi_{sz}}^{2})-{\dot{q}}_{m}(t-\eta_{\chi_{sz}}^{2}))-K_{P}(q_{s}(t-\eta_{\chi_{sz}}^{1})-q_{m}(t-\eta_{\chi_{sz}}^{1})) \end{array}$$

**Remark** **2.** *Due to the symmetric dynamics of the master and slave robots, controller gains are assumed the same in the bilateral structure*.

## 3. Sensor Allocation with Exponential Stability Analysis

This section provides a basis for sensor partitioning to guarantee the exponential stability of the sampled-data system. According to the previous section, it is assumed that four sensors with various sampling rates are available, thus $n_{s}=4$.

Data-drop out is modelled via switches in Figure 2.; let nominate sensing blocks by $S_{i},i\in IS$ where $IS=\{1,\ldots ,n_{s}\}$. We define $P=\{IS_{i}|i\in IS\}$ as a partition of sensors with the following properties:

(14) $$\begin{array}{l} IS_{i}\neq \emptyset ,\forall i\in IS\\ IS_{i}\cap IS_{i^{\prime }}=\emptyset ,\forall i\neq i^{\prime }\\ \cup IS_{i}=\{1,\ldots ,n_{x}\}. \end{array}$$

Each sensor in $S_{i},i\in IS$ is dedicated to sample one of the transmitted signals on both sides of the system. To parameterize the partition matrix, we define $Y\in {\left\{0,1\right\}}^{n_{x}\times n_{s}}$; its rows correspond to the states, as well as the columns to the $n_{s}$ sensors, belonging to the set of $IS_{i},i\in IS$. The matrix’s elements are defined in the Boolean set {0,1}. When Y(i,j)=1; it means that the *j*-*th* state is sampled by *i*-*th* sensor or, in other words, $j\in IS_{i}$; in the case of Y(i,j)=0, we have $j\notin IS_{i}.$ The maximum admissible sampling periods are known and named by $\sigma_{i}$ where i∈IS. To imply this partitioning to the multi-rate controller the diagonal matrices $C_{i}\in {\left\{0,1\right\}}^{n_{x}\times n_{x}},i\in IS$ is considered as:

(15) $$C_{i}(j,j)=\left\{ \begin{array}{l} 1\;if\;j\in IS_{i}\\ 0\;otherwise \end{array}\right.$$

It is presumed that sensors have a time-driven structure, and ZOHs are event-driven and update themselves immediately after receiving new data.

The following proposed theorem provides LMIs to assign state vectors to each sensor and assures that the stability does not jeopardize.

**Theorem** **1.** *Consider the sampled-data dynamic of the teleoperation system in (6) and (13). For positive scalars α and β and positive definite matrices*E$W_{o},N,\bar{Q},Q$*, and*$W_{i}(i\in IS)$*, there exists a sensor network parametrized by a matrix* Y*that guarantees the exponential stability with a rate greater than* σ/2*, if it satisfies the following LMIs:*

(16) $$\left[\begin{array}{ccc} \Gamma_{1}+\sigma \Gamma_{2} & * & *\\ \Lambda \sum_{i\in IS}W_{i} & -\sum_{i\in IS}\frac{W_{i}}{\sigma_{i}} & *\\ {\bar{Q}}^{T} & 0 & -\sum_{i\in IS}\frac{W_{i}}{\sigma_{i}e^{\alpha \sigma_{i}}} \end{array}\right]<0$$

(17) $$\left[\begin{array}{cccc} \Gamma_{1}+\sigma \Gamma_{3} & * & * & *\\ \Lambda \sum_{i\in IS}W & \sum_{i\in IS}\frac{W_{i}}{\sigma_{i}} & * & *\\ {\bar{R}}^{T} & 0 & -\sum_{i\in IS}\frac{W_{i}}{\sigma_{i}e^{\alpha \sigma_{i}}} & *\\ R^{T}\sigma & 0 & 0 & -\sigma e^{-\alpha \sigma }W_{0} \end{array}\right]<0$$

*where*

(18) $$\Lambda =\left[A_{\chi }\;-B_{\chi }K_{p}\;-B_{\chi }(K_{v})\;B_{\chi }K_{p}\;B_{\chi }K_{v}\;\underline{0}\right]$$

(19) $$\begin{array}{l} \Gamma_{1}=\Lambda^{T}[E\;\underline{0}\;\underline{0}]+\sigma \left[\begin{array}{c} I\\ 0\\ 0 \end{array}\right]E{\left[\begin{array}{c} I\\ 0\\ 0 \end{array}\right]}^{T}+\left[\begin{array}{c} E\\ 0\\ 0 \end{array}\right]\Lambda -\left[\begin{array}{ccc} I & \underline{0} & -I\\ \underline{0} & \underline{0} & \underline{0}\\ \underline{0} & \underline{0} & \underline{0} \end{array}\right]Q^{T}\\ -Q\left[\begin{array}{ccc} I & \underline{0} & -I\\ \underline{0} & \underline{0} & \underline{0}\\ \underline{0} & \underline{0} & \underline{0} \end{array}\right]-\left[\begin{array}{c} I\\ -I\\ 0 \end{array}\right]{\bar{Q}}^{T}-\left[\begin{array}{c} I\\ 0\\ -I \end{array}\right]N\left[I\;0\;-I\right]-\bar{Q}[I\;-I\;\underline{0}] \end{array}$$

(20) $$\begin{array}{l} \Gamma_{2}=\left[\Lambda \;0\right]W_{0}\left[\begin{array}{ccc} I & 0 & 0\\ 0 & 0 & I \end{array}\right]+{\left[\begin{array}{c} \Lambda \\ I\\ \underline{0} \end{array}\right]}^{T}Q_{0}\left[\begin{array}{c} \Lambda \\ I\\ \underline{0} \end{array}\right]+\Lambda^{T}N[I\;0\;-I]+\left[\begin{array}{c} I\\ 0\\ -I \end{array}\right]N\Lambda \\ +\sigma {\left[I\;0\;-I\right]}^{T}\;N[I\;0\;-I]\\ \Gamma_{3}=-{\left[\begin{array}{cccc} 0 & 0 & 0\\ 0 & I & 0 & \\ 0 & 0 & I \end{array}\right]}^{T}Q^{T}-Q\left[\begin{array}{ccc} 0 & 0 & 0\\ 0 & I & 0\\ 0 & 0 & I \end{array}\right] \end{array}$$

**Proof.**  To verify the theorem above, we present an LKF function and show that the LMIs in (16) and (17) satisfy the stability preliminaries. Let us define the Lyapunov function as:

(21) $$V(t,x_{t})=V_{1}+V_{2}+V_{3}+V_{4}$$

where

(22) $$\begin{array}{l} V_{1}=x^{T}(t)Ex(t)\\ V_{2}=(\sigma_{z}-\eta_{z})\int_{t-\eta_{z}}^{t}e^{\sigma (s-t)}[{\dot{x}}^{T}(s){\bar{x}}^{T}(s)x^{T}(z_{k})]W_{0}{\left[{\dot{x}}^{T}(s){\bar{x}}^{T}(s)x^{T}(z_{k})\right]}^{T}ds\\ V_{3}=\sum_{i=IS}\left(\sigma_{i}-\eta_{i}\right)\int_{t-\eta_{i}}^{i}e^{\alpha (s-t)}{\left(C_{i}\dot{x}(s)\right)}^{T}\bar{W}(C_{i}\dot{x}(s))ds\\ V_{4}=(\sigma_{z}-\eta_{z})[\;x^{T}(t)\;x^{T}(z_{k})]N{\left[x^{T}(t)\;x^{T}(z_{k})\right]}^{T} \end{array}$$

We demonstrate that LMIs in Theorem 1 are sufficient conditions for $\dot{V}+\alpha V\leq 0$ in the period among two successive sampling rates. The derivative of $V_{1},V_{2}$ and $V_{4}$ are straightforward and can be calculated as:

(23) $${\dot{V}}_{1}={\dot{x}}^{T}Ex+x^{T}E\dot{x}$$

(24) $$\begin{array}{l} {\dot{V}}_{2}=-\int_{t-\eta_{z}}^{t}e^{\sigma (s-t)}[{\dot{x}}^{T}(s)\;{\bar{x}}^{T}(s)\;x^{T}(z_{k})]W_{0}{\left[{\dot{x}}^{T}(s)\;{\bar{x}}^{T}(s)\;x^{T}(z_{k})\right]}^{T}ds\\ +(\sigma_{z}-\eta_{z})[{\dot{x}}^{T}\;{\bar{x}}^{T}\;x^{T}(z_{k})]W_{0}{\left[{\dot{x}}^{T}\;{\bar{x}}^{T}\;x^{T}(z_{k})\right]}^{T}-\sigma V_{2} \end{array}$$

(25) $$\begin{array}{l} {\dot{V}}_{4}=-[x^{T}\;x^{T}(z_{k})]N{\left[x^{T}\;x^{T}(z_{k})\right]}^{T}+(\sigma_{z}-\eta_{z})[{\dot{x}}^{T}\;0]N{\left[x^{T}\;x^{T}(z_{k})\right]}^{T}\\ +(\sigma_{z}-\eta_{z}){\left[x^{T}\;x^{T}(z_{k})\right]}^{T}N[{\dot{x}}^{T}\;\underline{0}] \end{array}$$

Considering $t-\eta_{z}=t_{n}$ for an arbitrary vector k(t) ${\dot{V}}_{2}$ can be simplified as:

(26) $$\begin{array}{l} {\dot{V}}_{2}\leq \eta_{z}k^{T}e^{\alpha \sigma_{z}}W_{0}^{-1}k-[x^{T}-{\bar{x}}^{T}{(z}_{k})\;\eta_{z}{\;x\;¯}^{T}\eta_{z}x^{T}(z_{k})]k-k^{T}[x^{T}-x^{T}{(z}_{k})\;\eta {\bar{x}}^{T}\eta x^{T}(z_{k})]+\\ (\sigma_{z}-\eta_{z})[{\dot{x}}^{T}{\bar{x}}^{T}x^{T}(z_{k})]W_{0}{\left[{\dot{x}}^{T}{\bar{x}}^{T}x^{T}(z_{k})\right]}^{T}-\alpha V_{2} \end{array}$$

A similar inequality can be assigned for the derivative of $V_{3}$ as follows:

(27) $$\begin{array}{l} {\dot{V}}_{3}\leq \sum_{i=IS}((C_{i}k_{i}{)}^{T}e^{\alpha \sigma_{i}}{\bar{W}}^{-1}\sigma^{i}(C_{i}k_{i})+{\left(C_{i}\dot{x}\right)}^{T}\bar{W}({\bar{W}}^{-1}\sigma^{i}\bar{W})(C_{i}\dot{x}))\\ -{\left(\Delta \bar{x}\right)}^{T}\bar{k}-{\bar{k}}^{T}(\Delta \bar{x})-\alpha V_{3} \end{array}$$

where $\bar{W}=\sum_{i\in IS}W_{i}$ and $\bar{x}(t)=\sum_{i\in IS}C_{i}x(t-\eta_{i}(t))$.

Let define the augmented parameters as:

(28) $$\Omega (t)={\left[x_{m}^{T}\;{\bar{x}}_{m}^{T}\left(t\right)\;x_{m}^{T}(z_{k})\;x_{s}^{T}\;{\bar{x}}_{s}^{T}\left(t\right)\;x_{s}^{T}(z_{k})\right]}^{T}$$

Thus:

(29) $$\dot{x}(t)=\left[A_{i}\;-B_{i}K_{p}\;-B_{i}(K_{v})\;B_{i}K_{p}\;B_{i}K_{v}\;\underline{0}\right]\Omega (t)$$

choosing $k(t)=Q^{T}(t)\Omega (t)$ and $\bar{k}(t)={\bar{Q}}^{T}(t)\Omega (t)$ and substituting (23)–(27) in $\dot{V}+\alpha V\leq 0$ yields:

(30) $$\begin{array}{l} \dot{V}+\alpha V={\dot{V}}_{1}+{\dot{V}}_{2}+{\dot{V}}_{3}+{\dot{V}}_{4}+\alpha (V_{1}+V_{2}+V_{3}+V_{4})\leq \Omega^{T}({\left[I\;\underline{0}\;\underline{0}\right]}^{T}E\Lambda +\Lambda^{T}E[I\;\underline{0}\;\underline{0}]\\ +\alpha {\left[I\;\underline{0}\;\underline{0}\right]}^{T}E[I\;\underline{0}\;\underline{0}]+\eta Qe^{\alpha \sigma }W_{0}^{-1}Q^{T}-\left[\begin{array}{ccc} I & \underline{0} & -I\\ \underline{0} & \eta I & \underline{0}\\ \underline{0} & \underline{0} & \eta I \end{array}\right]W^{T}-W\left[\begin{array}{ccc} I & \underline{0} & -I\\ \underline{0} & \eta I & \underline{0}\\ \underline{0} & \underline{0} & \eta I \end{array}\right]\\ +(\sigma -\eta ){\left[\begin{array}{l} \Lambda \\ I\\ \underline{0} \end{array}\right]}^{T}W_{0}\left[\begin{array}{l} \Lambda \\ I\\ \underline{0} \end{array}\right]+\bar{Q}(\sum_{i\in IS}\frac{W_{i}}{\sigma_{i}})-\left[\begin{array}{l} I\\ -I\\ 0 \end{array}\right]{\bar{Q}}^{T}-\bar{Q}[I-I\underline{0}]+\Lambda^{T}\bar{W}{\left(\sum_{i\in IS}\frac{W_{i}}{\sigma_{i}}\right)}^{-1}\bar{W}\Lambda \\ +(\alpha (\sigma -\eta )-1)\left[\begin{array}{l} I\\ 0\\ -I \end{array}\right]N\left[I\;0\;-I\right]+(\sigma -\eta )({\left[\begin{array}{c} \Lambda \\ \underline{0} \end{array}\right]}^{T}N\left[I\;0\;-I\right]+\left[\begin{array}{l} I\\ 0\\ -I \end{array}\right]N\left[\begin{array}{c} \Lambda \\ \underline{0} \end{array}\right])\Omega \end{array}$$

Applying Schur complement, by substituting η=0 and η=σ, the LMIs (16) and (17) implies $\dot{V}+\sigma V<0$. Since (30) is affine in η, LMIs (16) and (17) are held for any η∈(0,σ) and satisfy the condition for Theorem 1, completing the proof. □

Based on the theorem above, the problem of state assignments for sensors that satisfies the stability can be restated as finding $Y\in {\left\{0,1\right\}}^{4*4}$ subject to the $E>0,W_{0}>0,N>0,W_{i}=W_{i}^{T},i\in IS,\alpha >0,\beta >0$ and LMIs (16) and (17).

## 4. Observer Design for the Multi-Rate Teleoperator System

**Figure 3:**
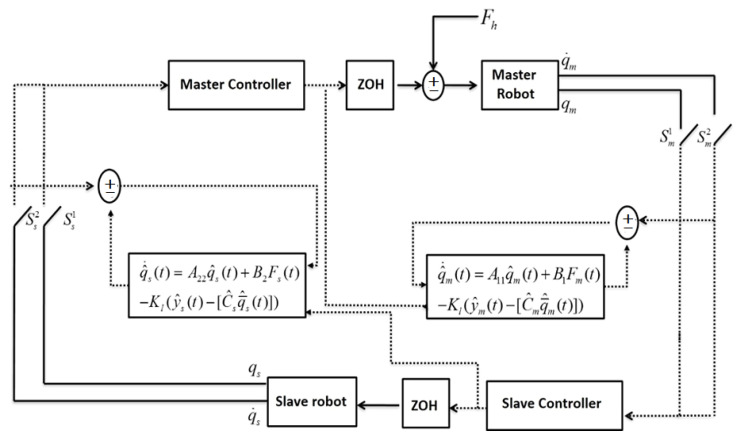
Schematic of the multi-rate observer for the teleoperator system.

In this section, observer design for the multi-rate teleoperator system has been addressed. For the calculated maximum admissible sampling periods, the contribution of this section is providing Krasovskii-based conditions to design linear observers on both sides of the teleoperator system. The estimation error of the observer should converge to the origin exponentially. The extension schematic of the sampled-data system with multi-rate observers has been depicted in Figure 3.

In this scheme, $K_{l}$ is the gain of Luenberger observer. $A_{11},A_{22},B_{1}$ and, $B_{2}$ have been defined in (3) and (4), respectively. The estimation error of the observer is notified by $e(t)=q(t)-\hat{q}(t).$ Thus, the error dynamics can be defined as:

(31) $$\begin{array}{l} \hat{q}(t)=A\hat{q}(t)-K_{l}(y(t)-\hat{C}\hat{q}(t))+Bu(t)\\ \dot{e}(t)=(A+K_{l}C)e(t) \end{array}$$

where:

(32) $$A=\left[\begin{array}{cc} A_{11} & \underline{0}\\ \underline{0} & A_{22} \end{array}\right]\;,\;B=\left[\begin{array}{c} B_{1}\\ B_{2} \end{array}\right]$$

Thus, Theorem 1 can be extended to calculate the observer gain via provided LMIs.

**Theorem** **2.** *Given* $\sigma_{x}^{i}i\in (1,2,3,4)$ *and*  α>0 *, there exists an observer gain* $K_{l}=E^{-1}Y$ *which the estimation error of the observer is exponentially stable for that if there exist positive definite matrices* E *and* $W_{0}$ *and matrices* Y,Q *and* $\bar{Q}$ *, with proper dimensions, satisfying the following LMIs:*

(33) $$\left[\begin{array}{cccc} \Gamma_{1}+\sigma \Gamma_{2} & * & * & *\\ \left[\begin{array}{lll} EA & & \left[Y\bar{C}0\right]\\ 0 & W_{0} \end{array}\right] & -\sigma diag(E,W_{0}) & * & *\\ \bar{F}\sigma {\bar{Q}}^{T} & 0 & -\bar{F}\sigma \bar{W} & *\\ \left[EAY\bar{C}0\right] & 0 & 0 & -\bar{W}\sigma \end{array}\right]<0$$

(34) $$\left[\begin{array}{cccc} \Gamma_{1}+\sigma \Gamma_{3} & * & * & *\\ \bar{F}\sigma {\bar{Q}}^{T} & -\bar{F}\sigma \bar{W} & * & *\\ \left[EA\;Y\bar{C}\;0\right] & 0 & -\bar{W}\sigma & *\\ Q^{T} & 0 & 0 & \frac{-\sigma W_{0}}{\exp(\alpha \sigma )} \end{array}\right]<0$$

*where* $\Gamma_{1},\Gamma_{2}$ *and,* $\Gamma_{3}$ *were defined in (19) and (20) and* $\bar{F}=diag(\exp(\alpha \sigma^{i})I)\;i\in (1,2,3,4)$

**Proof.**  The proof is straightforward and similar to the proof of Theorem 1. It is enough to replace the Lyapunov function in (21) with the following terms and proceed with the same steps in Theorem 1.

(35) $$\begin{array}{l} V_{1}=e^{T}(t)Ee(t)\\ V_{2}=(\sigma_{z}-\eta_{z})\int_{t-\eta_{z}}^{t}e^{\sigma (s-t)}[{\dot{e}}^{T}(s){\bar{e}}^{T}(s)e^{T}(z_{k})]W_{0}{\left[{\dot{e}}^{T}(s){\bar{e}}^{T}(s)e^{T}(z_{k})\right]}^{T}ds\\ V_{3}=\sum_{i=IS}\left(\sigma_{i}-\eta_{i}\right)\int_{t-\eta_{i}}^{i}e^{\alpha (s-t)}{\left(C_{i}\dot{e}(s)\right)}^{T}\bar{W}(C_{i}\dot{e}(s))ds\\ V_{4}=(\sigma_{z}-\eta_{z})[\;e^{T}(t)e^{T}(z_{k})]N{\left[e^{T}(t)e^{T}(z_{k})\right]}^{T} \end{array}$$

□

The theorem above provides sufficient conditions for exponential convergence of estimation error to the origin for the multi-rate observer. It is assumed that the upper bound of sampling intervals for all sensors is available. The aim is to design observers whose gains are calculated via an optimization process stated in the forms of LMIs. Indeed, we first find Y subject to E>0 and $W_{0}>0$, and then compute the observer gain as $K_{l}=E^{-1}Y$.

## 5. Numerical Simulation

This section illustrates the circumstance of states assignment between sensors for the teleoperator system via numerical simulations. Two DOF robotic manipulators are considered as master and slave subsystems with the following dynamic parameters:

(36) $$M_{m}=M_{s}=3.5,\;D_{m}=D_{s}=2.5,\;S_{m}=S_{s}=0.3$$

**Figure 4:**
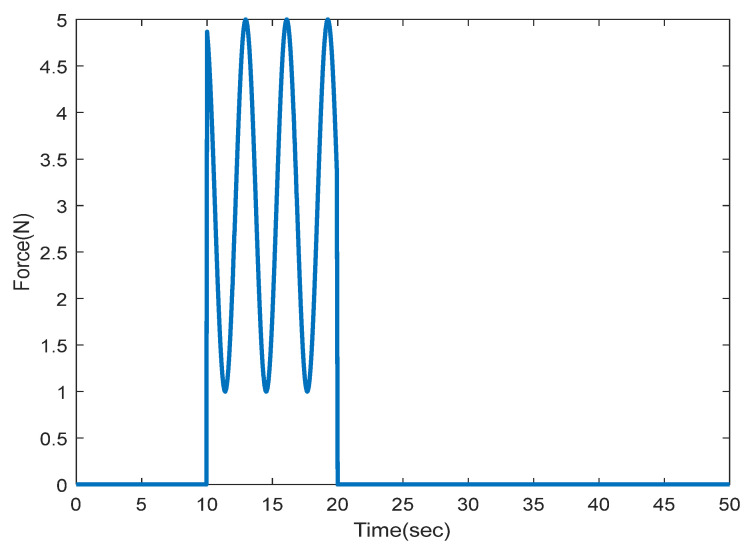
Exerted force to the master robot by the operator.

The exerted force is modelled by mass-spring dynamics with 15 N/m for spring gain and 2Ns/m for the damper gain; this force is depicted in Figure 4. It is assumed that the slave robot’s end-effecter has touched the environment at 2rad, as well as the controller gains are set to $K_{p}=15$ and $K_{v}=0.3$. The tuning process is based on the method proposed in [25]. In this approach, the integral of the absolute error is considered a cost function for the optimization algorithm to find optimal gain values for the PD controller to achieve the best tracking performance. According to Theorem 1, the sensor partitioning problem that preserves the stability can be transformed into a mixed-integer program with LMIs. This last is in contrast with our previous study, where the maximum admissible sampling periods and refresh rates had been calculated.

**Figure 5:**
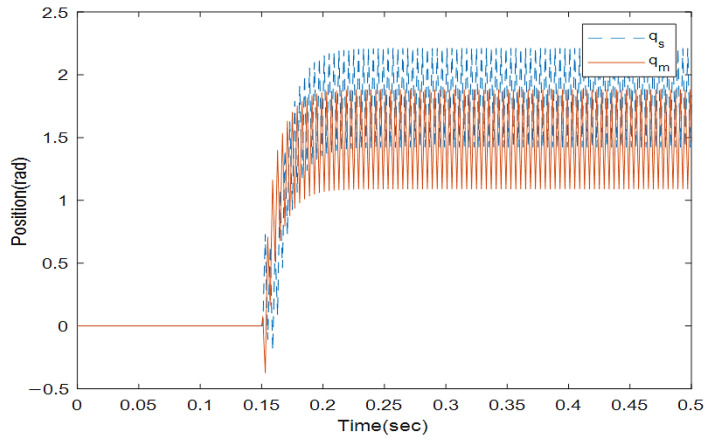
Position trajectories with random sensor partitioning.

**Figure 6:**
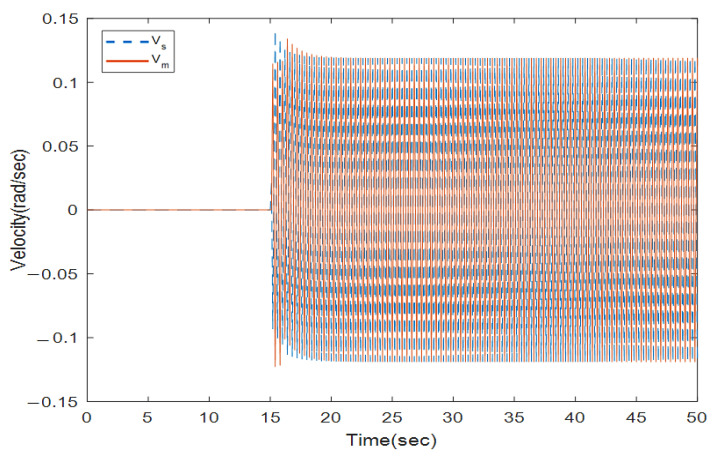
Velocity trajectories with random sensor partitioning for the master (V_m_) and slave (V_s_) robots.

**Figure 7:**
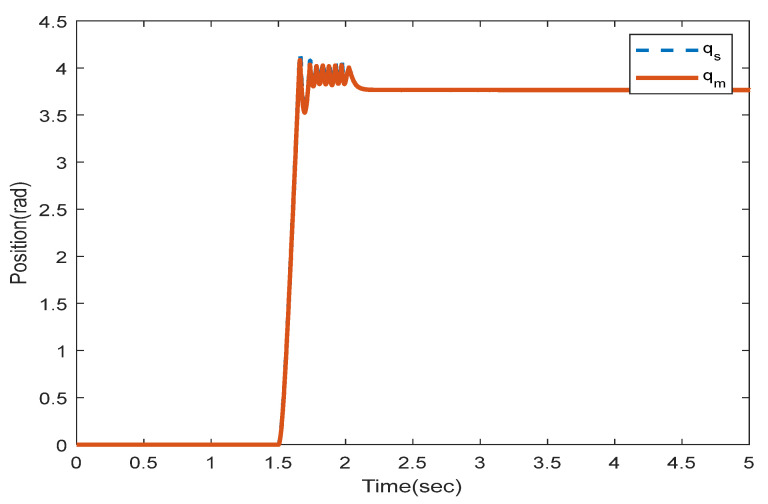
Position trajectories utilizing two encoders with multi-rate sampling related to sensor allocation LMIs in Theorem 1.

**Figure 8:**
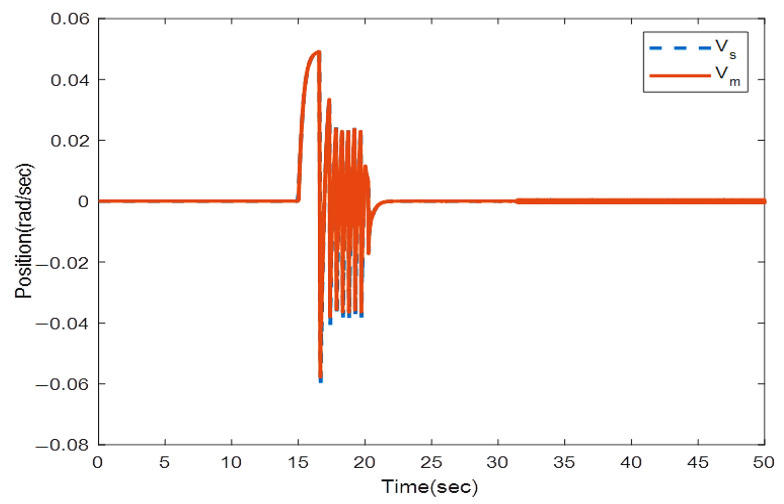
Velocity trajectories utilizing two encoders with multi-rate sampling related to sensor allocation LMIs in Theorem 1.

Two scenarios are defined to evaluate the sensor partitioning problem. In the first scenario, the master robot position signal for the first state and second state is presumed to be acquired via encoders at sampling periods up to 0.4 s and 0.6 s, and the rate of the velocity states on both sides will be measured by inertial measurement unit is 0.8 s. Thus, with master robot position signals and master-slave robots velocity signals assigned to the sensors, the sensor network matrix *Y* is defined as:

(37) $$Y=\left[\begin{array}{ll} 1 & 0\\ 0 & 1\\ y_{1} & y_{2}\\ y_{3} & y_{4} \end{array}\right]$$

where the rows of this matrix indicate position signals for two links. The first column is related to the encoder with a 0.4 s sampling rate and the second column with a 0.6 s sampling rate. We solve the LMIs in Theorem 1 to find which encoder should be utilized to sample the position signals in the slave side such that the stability is preserved. Solving (16) and (17) with the MATLAB toolbox YALMIP [26] yields: $y_{1}=1,y_{2}=0,y_{3}=0$, and $y_{4}=1$. This means that the first and second position states of the slave robot should be assigned to the first encoder and the second encoders, respectively, to provide exponential stability. To validate this concept, the position and velocity trajectories are depicted in Figure 5, Figure 6, Figure 7 and Figure 8.

Figure 5 and Figure 6 show the instability of the teleoperator system when the second encoder is utilized for the first position state of the slave robot side. In Figure 7 and Figure 8, both teleoperator systems’ stability and transparency are obtained considering the proper sensor partitioning on the slave robot side.

The root mean squared error (RMSE) is the square root of the quadratic mean of all errors. The calculated RMSE error related to Figure 8 is equal to 0.0003416.

To illustrate the efficiency of multi-rate sampling in comparison with the single-rate sampling, the maximum admissible sampling period based on the LMIs in Theorem 1 is calculated with the YALMIP software [26] and demonstrated in the following Table 1.

It is deduced that by applying multi-rate samplers, there will be fewer conservative conditions for sampling instants or update rates to guarantee the system’s stability. Specifically, while the maximum admissible sampling period for position sensor to preserve the stability is 150 ms in the single-rate method, multi-rate architecture can increase this upper bound to 352 ms.

The second scenario assumes that the transmitted signals are assigned to sensors with different and nonuniform sampling rates. These sensors can be categorized as a fast-rate sensor with a maximum sampling interval of 0.2 s, a medium-rate sensor with a maximum sampling interval of 0.6 s and a slow-rate sensor with a maximum sampling interval of 1 s. The aim is to assign these sensors to the transmitted signals on both sides, preserving stability. In this scenario, the sensor network matrix should have eight rows for four position and four-velocity signals and three columns for fast, medium and slow rate sensors. Thus, the number of Boolean elements stored in Y is 24. Solving LMIs in Theorem 1, the sensor network matrix *Y* is defined as:

(38) $$Y={\left[\begin{array}{l} 1\;1\;0\;0\;1\;1\;0\;0\\ 0\;0\;1\;1\;0\;0\;1\;1\\ 0\;0\;0\;0\;0\;0\;0\;0 \end{array}\right]}^{T}$$

**Figure 9:**
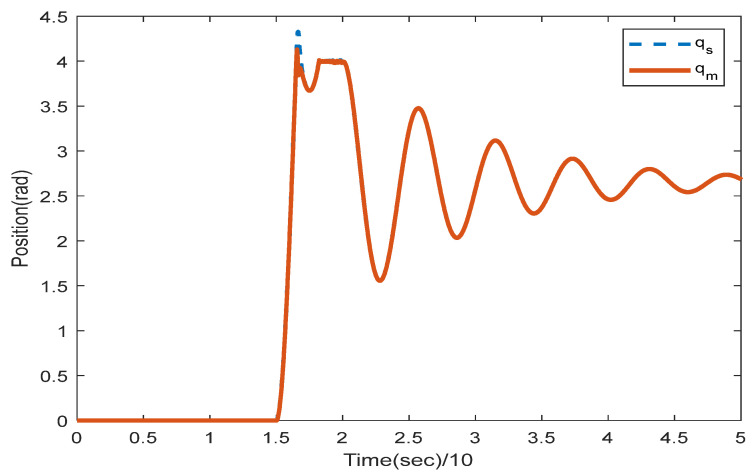
Position trajectories for sensor allocation scheme provided in scenario 2 related to LMIs in Theorem 1.

**Figure 10:**
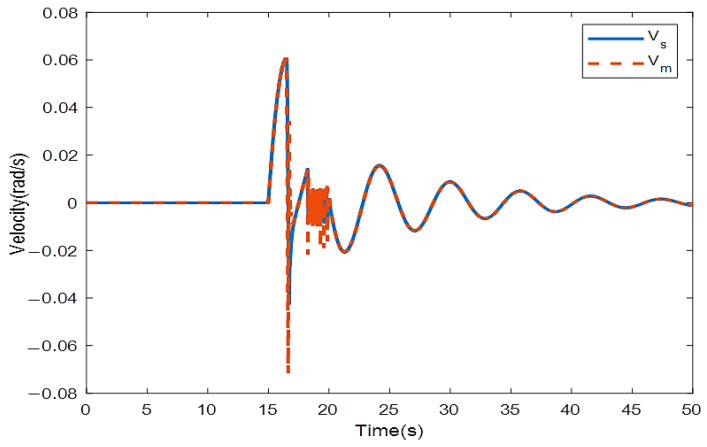
Velocity trajectories for sensor allocation scheme provided in scenario 2 related to LMIs in Theorem 1.

In the proposed sensor allocation scheme, position signals are sampled with the fast-rate samplers according to their significant roles in stabilizing the system. Position and velocity trajectories for this case are depicted in Figure 9 and Figure 10. The bilateral system becomes unstable if slow-rate sensors are used to sample the position signals.

The calculated RMSE is equal to 0.0002855 for position trajectories related to the scenario 2 and 0.000965 for velocity trajectories.

Now, suppose that the first position states of the master and slave robots are measured with an encoder with an unknown sampling period smaller than 0.4 s, and the second position states are measured with an encoder with a maximum permissible sampling period of 0.6 s. Solving the problem defined in Theorem 2 for α=0.2 lead to the following observer gain:

(39) $$K_{l}=\left[\begin{array}{l} -0.7201\;-0.1154\\ -0.3022\;-0.1125\\ -0.8251\;-0.6215\\ -0.8894\;-0.7212 \end{array}\right]$$

**Figure 11:**
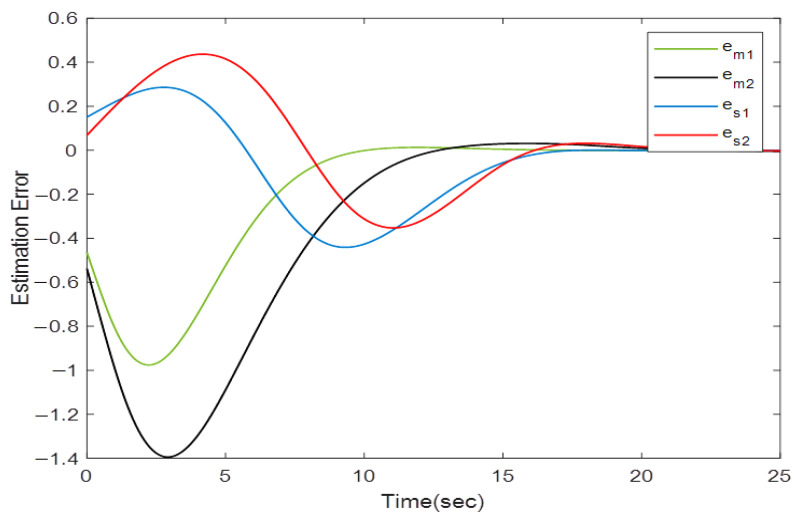
Position estimation error trajectory for observer gain calculated in (39).

The position estimation errors for the teleoperator system are depicted in Figure 11, which confirms that the observer gain in (39) preserves the exponential stability of the estimation error.

## 6. Discussion

In this paper, the sensor allocation approach is designed for sampled-data bilateral teleoperation systems. Based on Theorem 1 and its proof, this problem is converted into a mixed-integer program with LMIs that, if satisfied, the teleoperation system’s stability will be preserved. The solution is stated as a sensor network matrix, that its rows represent the transmitted states and its columns show the number of sensors with varying sampling rates. The elements of this matrix are defined in the Boolean set {0,1}, where 1 means that the state is sampled by the related sensor.

In contrast to in-depth studies on continuous-time teleoperation models in the literature, there is less research on sampled-data teleoperation systems. However, as deduced from the results and simulations in Section 5, sampling has a significant impact on the stability and neglecting it can undermine both transparency and stability of the teleoperator systems. Compared to similar studies that take the sampling analysis into account and provide a sampled-data model for teleoperator systems such as [19,27,28], the novelty of the proposed method relies on designing a sensor allocation basis for the multi-rate structure of bilateral teleoperator systems. This proposed method covers the significant deficiency in previous literature of discrete-time teleoperations systems, where all transmitted signals are sampled with the same sampling rates. Uniform sampling is not sensible for teleoperation systems since, besides the possibility of utilizing various sensors with varying sampling rates on both sides, master and slave robots may have different hardware restrictions regarding the existence distance. Therefore, a multi-rate approach has been presented in this study to collect data through numerous sensors with various sampling rates, as an extension to our previous investigations, where the maximum sampling interval of sensors has been calculated to guarantee the stability of the teleoperator system. Indeed, in [29], we introduced a stability framework for single-rate uniform and nonuniform sampling intervals for teleoperator systems. Also, in [22], the transmission rates’ upper bounds were calculated to guarantee the teleoperator system stability. However, the sensor allocation problem among different possible scenarios was an open question covered in the proposed research work. For this purpose, an additional term is added to Lyapunov-Krasovskii function in (21) based on the diagonal matrix C to consider the multi-rate partitioning of sensors. Thus, the stability concept is stated in LMIs, whose solution yields the sensor network matrix. It is concluded that stability can be guaranteed with higher admissible sampling intervals and less determinative conditions. Compared to other LMI schemes, such as input delay in [24], the LMIs presented in Theorem 1 impose less determinative conditions on the maximum permissible sampling interval regarding the defined Lyapunov-Krasovskii function.

Finally, the contributions of this study can be summarized as follows:

1. Designing a sensor allocation structure for bilateral teleoperator systems.
2. Providing exponential stability analysis for the proposed multi-rate sampling approach to collect data through numerous sensors with various sampling rates in teleoperation systems.
3. Designing linear observers for master and slave robots sides of sampled-data teleoperation systems relied on Krasovskii-based conditions and maximum admissible sampling periods.

These evaluations can aid in the development of guidelines for more transparent and stable teleoperator systems.

## 7. Conclusions

Unlike continuous-time teleoperation systems, there are far fewer studies for sampled-data counterparts. A significant deficiency in previous literature on discrete-time teleoperations systems is that all transmitted signals are sampled with the same sampling rate. This study has investigated the effect of sampling rates of samplers and update rates of ZOHs on the teleoperator system’s stability. Variable sampling intervals were considered for transmitted signals due to the isolation of the master and slave robots and inner time delays and data dropout in the communication channel. Also, in this paper, the sensor allocation issue was addressed for preserving the exponential stability of the sampled-data teleoperator systems using multi-rate sampling. This structure leads to less conservative conditions in the sense of stability and maximum admissible sampling periods. Sufficient conditions based on the Krasovskii functions were utilized to partition the transmitted signals according to the provided LMIs. Besides, these conditions were employed to design observers for determining the maximum admissible sampling periods for each sensor. As future works, these results can be extended to four-channel teleoperator systems with nonlinear dynamics.

## Figures and Tables

**Table 1:** Comparison of maximum admissible sampling rates for single-rate and multi-rate architectures.

Single-Rate Sampling		$\sigma_{j}^{2}$
$\sigma_{j}^{1}=150\;\mathrm{ms}$	$\sigma_{z}=150\;\mathrm{ms}$	150 ms
**Multi-Rate Sampling**		
$\sigma_{j}^{1}=40\;\mathrm{ms}$	$\sigma_{z}=600\;\mathrm{ms}$	352 ms

## Data Availability

Data of our study are available upon request.
